# Effective Removal of Sulfanilic Acid From Water Using a Low-Pressure Electrochemical RuO_2_-TiO_2_@Ti/PVDF Composite Membrane

**DOI:** 10.3389/fchem.2018.00395

**Published:** 2018-09-06

**Authors:** Junjian Zheng, Kaili Yan, Zhichao Wu, Mingxian Liu, Zhiwei Wang

**Affiliations:** ^1^State Key Laboratory of Pollution Control and Resource Reuse, School of Environmental Science and Engineering, Shanghai Institute of Pollution Control and Ecological Security, Tongji University, Shanghai, China; ^2^School of Chemical Science and Engineering, Tongji University, Shanghai, China

**Keywords:** sulfanilic acid, membrane filtration, degradation, wastewater treatment, electrochemical oxidation

## Abstract

Removal of sulfanilic acid (SA) from water is an urgent but still challenging task. Herein, we developed a low pressure electrochemical membrane filtration (EMF) system for SA decontamination using RuO_2_-TiO_2_@Ti/PVDF composite membrane to serve as not only a filter but also an anode. Results showed that efficient removal of SA was achieved in this EMF system. At a charging voltage of 1.5 V and a electrolyte concentration of 15 mM, flow-through operation with a hydraulic retention time (HRT) of 2 h led to a high SA removal efficiency (80.4%), as expected from the improved contact reaction of this compound with ROS present at the anode surface. Cyclic voltammetry (CV) analysis indicated that the direct anodic oxidation played a minor role in SA degradation. Electron spin resonance (ESR) spectra demonstrated the production of ^•^OH in the EMF system. Compared to the cathodic polarization, anodic generated ROS was more likely responsible for SA removal. Scavenging tests suggested that adsorbed ^•^OH on the anode (>^•^OH) played a dominant role in SA degradation, while ${\text{O}}_{2}^{•-}$ was an important intermediate oxidant which mediated the production of ^•^OH. The calculated mineralization current efficiency (MCE) of the flow-through operated system 29.3% with this value much higher than that of the flow-by mode (5.1%). As a consequence, flow-through operation contributed to efficient oxidation of SA toward CO_2_ and nontoxic carboxylic acids accounting for 71.2% of initial C. These results demonstrate the potential of the EMF system to be used as an effective technology for water decontamination.

## Introduction

Sulfanilic acid (SA) is one of the most widely-used sulfonated aromatic amines in the production of azo dyes, dyeing auxiliaries, food coloring, pharmaceuticals perfumes and pesticides (Chen et al., 2012; El-Ghenymy et al., 2012). With the extensive applications of SA, the release of SA into water environment is unavoidable due to the disposal of SA-bearing waste, discharge of wastewater and surface runoff, which will threaten environmental health and water safety in view of the inherent characteristics of SA including high polarity, large water solubility and high chemical stability (nonbiodegradability) (Riu et al., 1997). The resulting water contamination calls for the development of cost-effective treatment technologies.

Low-pressure membrane filtration, e.g., microfiltration (MF) and ultrafiltration (UF), offers an attractive technology for water/wastewater due to its small footprint, and high efficiency in removing bacteria and colloidal/particulate matter (Zimer et al., 2016). However, low-pressure membrane filtration is incapable of removing low molecular weight organic matter, e.g., SA, due to the size exclusion effects. Therefore, the combination/integration of other robust technologies with low-pressure membrane filtration process is needed to address this concern.

In recent years, electrochemical advanced oxidation process has attracted much attention for refractory organic matter removal (Radjenovic and Sedlak, 2015; Moreira et al., 2016) due to its high efficiency and relatively low energy consumption. In particular, the use of electrochemical oxidation under anodic polarization has been reported to be capable of eliminating a variety of micropollutants (Park et al., 2018), attributed to direct oxidation on the anode surface and/or indirect oxidation induced by reactive oxygen species (Antonin et al., 2015; Martínez-Huitle et al., 2015; Oturan et al., 2015; Trellu et al., 2016). Integration of anodic oxidation approach with membrane filtration process has been implemented by developing Ti_4_O_7_ membranes (Geng and Chen, 2017) and carbon-based membranes (Yang et al., 2012; Dudchenko et al., 2014). However, these bare filters suffer from drawbacks such as rigorous preparation conditions and, in particular, the potential fouling of electrodes caused by the deposition of colloids and particles which will significantly impair the anodic oxidation efficiency. In contrast, a composite electrochemical membrane can overcome above-mentioned disadvantages since membrane separation retains colloidal and particulate matters, thus protecting the anode activity.

RuO_2_-TiO_2_@Ti anode, as one of typical dimensionally stable anodes, which is known to have high efficacy in the degradation of refractory organics in contaminated waters, owing to the synergistic effects of binary metal oxides nanoparticles in the presence of external electric field (Yue et al., 2017; Li et al., 2018a; Park et al., 2018). The objective of this work was, therefore, to develop a low-pressure electrochemical MF membrane, i.e., RuO_2_-TiO_2_@Ti/polyvinylidene fluoride (PVDF) composite membrane. The performance in removing SA using this low-pressure composite membrane was investigated. Key questions to be addressed in the present work include (i) what about the electrochemical activity of the composite membrane? (ii) how do the operating conditions affect the removal behavior and oxidation efficiency of SA? (iii) what kinds of oxidants accounting for SA degradation?

## Materials and methods

### Materials

All chemicals were of analytical reagent grade and used without any further purification. Titanium mesh (pore size: 100 μm, and thickness: 50 μm) was obtained from Hebei Anheng company (China). Polyvinyli-dene fluoride (PVDF) materials were provided by Shanghai 3F (China). Polyethyleneglycole (PEG 600) and Dimethyl sulphoxide (DMSO) were obtained from Sinopharm (China). Sulfanilic acid (SA) (CAS number: 121-57-3) was purchased from Sigma-Aldrich (USA). Deionized water was used throughout the experiment. Solution pH was adjusted to 7 using 1 M H_2_SO_4_ or 1 M NaOH when necessary. 1 mM piperazine- N,N′-bis (ethanesulfonic acid) (PIPES) buffer was added to maintain a near-constant solution pH (pH = 7).

### RuO_2_-TiO_2_@Ti/PVDF membrane preparation

Titanium mesh was used in this study as the substrate of the electrode because of its corrosion resistance, good electrical conductivity and cost-effectiveness, as well as in view of the intrinsic drawbacks of the other metallic or non-metallic substrates, for instance, Ta, Zr, W, and Nb substrates are too expensive, while a Si substrate is quite brittle and has poor electrical conductivity (Chen and Nigro, 2003; Lin et al., 2012). Prior to use, the titanium mesh was first degreased in 10 wt% NaOH, polished with emery paper, and then etched in 10 wt% boiling oxalic acid solution for 0.5 h. The pretreated titanium mesh was washed clean with deionized water, and dried for use.

RuO_2_-TiO_2_@Ti electrode was prepared via the sol-gel method followed by coating technique. TiO_2_ sol-gel was synthesized using tetrabutyl titanate as the precursor (Yang et al., 2012). Tetrabutyl titanate (43 mL) was added to 88 mL of ethanol, and the solution was stirred for 0.5 h. Another 44 mL of ethanol was mixed with 18 mL of water and 8.9 mL of nitric acid. The ethanol/nitric acid/water solution was then slowly added into the tetrabutyl titanate solution. The resulting solution was stirred for 2 h, obtaining the transparent TiO_2_ sol. Solutions of RuCl_3_ were prepared using 100 mL 2-propanol as the solvent to dissolve 5.49 g RuCl_3_. Unless otherwise noted, the temperature was maintained at 25°C. The prepared TiO_2_ sol was coated onto the pretreated titanium mesh with a brush, and the painted titanium mesh was then dried at 100°C for 10 min. The coating process was repeated for 3 times, followed by calcination under 450°C for 30 min. After this, the electrode was coated with RuCl_3_ solutions, dried and calcined under the above conditions. These two steps were further repeated for 5 times and the final calcination step lasted for 2 h. The total loading amount of RuO_2_-TiO_2_ coating was ~0.24 g/m^2^.

The composite PVDF membrane was prepared by a phase inversion process. Membrane casting solution was prepared according to the procedure as documented elsewhere (Wang et al., 2012). Briefly, a predetermined amount of PVDF and PEG was dissolved in DMSO and agitated at 80°C for 48 h to form a homogeneous casting solution. After that, the homogeneous casting solution was casted on RuO_2_-TiO_2_@Ti electrode at a casting knife gap of 200 μm. The casted film was exposed to ambient air (20 ± 1°C, 30 ± 5% relative humidity) for 30 s to allow partial evaporation of the solvents and then immersed in a deionized water bath for phase-inversion at room temperature (25 ± 1°C) to form membrane pores.

### Membrane characterization

Surface morphologies of RuO_2_-TiO_2_@Ti electrode and the composite membrane were analyzed by scanning electron microscope with energy dispersive spectrometer (SEM/EDS) (Model XL-30, Philips, Netherland). Linear sweep voltammetry (LSV) and cyclic voltammetry (CV) scans were carried out using the neutral electrolyte solution containing 1 mM PIPES and 15 mM Na_2_SO_4_ (in some cases, 50 μM SA was added) in a three-electrode cell driven by an electrochemical workstation (CS350, Corrtest Co., China), to characterize the electrochemical properties of the developed RuO_2_-TiO_2_@Ti/PVDF composite membrane and clarify the relevant anodic oxidation mechanism of this membrane, respectively. An Ag/AgCl and a Pt wire served as the reference electrode and the counter electrode, respectively. In particular, in LSV tests, RuO_2_-TiO_2_@Ti electrode and pristine PVDF membrane were used as the control.

### Experimental setup

A schematic diagram of the electrochemical membrane reactor is shown in Figure S1. The reactor had an effective volume of 250 mL (5 × 5 × 10 cm) and was installed with two graphite plates (dimension = 5 × 5 cm; thickness = 0.5 cm) serving as the cathodes and the composite membrane module (dimension = 5 × 5 cm; effective area = 18 cm^2^) acting as the physical filter and anode. Two composite membranes were assembled on a bracket to form a membrane module. The distance between the anode and cathodes was 1 cm. The external electric field was supplied by a DC power supply (CHI1030C, Jiecheng, China) during the electrolysis experiments. A perforated plexiglas tube was mounted below the membrane module to supply air (or nitrogen gas if needed) and scour the membrane surface.

To investigate the performance of SA removal under different electric field strength, experiments were first conducted with applied voltages of 0, 0.5, 1.0, 1.5, and 2.0 V under flow-by mode, in which the influent and effluent pumps were turned off. Further experiments were performed under flow-through mode with the influent and effluent pumps switched on, in which the concentrations of oxidants (H_2_O_2_, reactive oxygen species (ROS) and •OH) were quantified. The membrane fluxes used in flow-through mode were 280, 140, 70, 35, and 28.3 L/(m^2^•h), resulting in a hydraulic retention time (HRT) of 0.5, 1, 2, 4, and 6 h (an equivalent time period to that used in the flow-by mode), respectively. Each experiment was initiated by adding 50 μM of SA (or, in some tests, probe compounds or scavengers were dosed) to 250 mL of air-saturated water (or, in some cases, water which had been deoxygenated by sparging with nitrogen) containing 1 mM PIPES buffer and 15 mM Na_2_SO_4_. The air or nitrogen gas was provided by an air pump with an aeration rate of 300 mL/min. The influence of electrolyte concentrations (Na_2_SO_4_) (0, 5, 10, 15 and 20 mM) on SA degradation was also investigated under flow-through mode. Samples extracted from the reaction cell (flow-by mode) and outlet of the membrane module (flow-through mode) were analyzed intermediately after filtration by 0.45 μm nylon syringe filters.

### Analytical methods

The concentrations of SA and its oxidation intermediates were measured using reversed-phase high-performance liquid chromatography (RP-HPLC, Agilent 1200, USA) and/or ion-exclusion HPLC (IEC, Agilent 1200, USA) or gas chromatography (GC, Agilent6890N, USA) with the detailed testing procedures as described in our previous work (Zheng et al., 2017). The concentration of aqueous H_2_O_2_ at the vicinity of membrane and cathode surface in the electrochemical system was determined using the metavanadate spectrophotometric method (Nogueira et al., 2005). In brief, 2 mL of samples collected from the reactor were immediately filtered and subsequently mixed with aliquots of H_2_SO_4_ and NH_4_VO_3_ stock solution in a quartz cuvette at ultimate H_2_SO_4_ and NH_4_VO_3_ concentrations of 58 mM and 6.2 mM, respectively, prior to their absorbance examination at UV detection wavelength of 450 nm on a TU-1810DPC spectrophotometer (PERSEE, Beijing, China). The total organic carbon (TOC) concentrations of samples were analyzed by a TOC analyzer (TOC-5000A, Shimadzu, Japan). Based on the TOC determination results, mineralization current efficiency (MCE) of the flow-by and flow-through system was evaluated according to the calculation protocols documented in Section S1.

The quantification experiments of ^•^OH and ROS generated in the flow-through operated system were performed in the dark using the neutral electrolyte solution containing 15 mM Na_2_SO_4_ without addition of SA in the influent. After 6 h operation of the system under different applied voltages, the reaction cell was dosed with excess probe compounds (1 mM 2,7-dichlorodihydrofluorescein diacetate (DCFH-DA) or 30 mM 5,5-dimethypylpyrroline-1-oxide (DMPO)). After filtration, the steady-state concentration of oxidation products of probe compounds in effluent was determined intermediately. DCFH-DA, which is known to non-selectively react with ROS (e.g., ^•^OH, ${\text{O}}_{2}^{•-}$/${\text{HO}}_{2}^{•}$, and H_2_O_2_) (Wang et al., 2017), was utilized in this study with its oxidation product (fluorescent DCF) quantified by a multi-mode microplate reader (Synergy 4, Bio-Tek Instruments, USA) with an excitation wavelength of 488 nm and emission wavelength of 525 nm. It has been well-established that the reaction between ^•^OH and DMPO leads to formation of hydroxyl radical adduct (DMPO-^•^OH) (Zhao et al., 2013b), DMPO was therefore chosen for ^•^OH examination in this study. Electron spin resonance (ESR) signals of DMPO-^•^OH were recorded on a Bruker EMX-10/12 spectrometer (Bruker, Karlsruhe, Germany) equipped with a Super-X microwave bridge and an irradiation source of Quanta-Ray ND:YAG laser system. The EPR parameters were as follows: microwave frequency, 9.853 GHz; microwave power, 20 mW; modulation amplitude, 1 G; modulation frequency, 100 kHz.

## Results and discussion

### Characterization of the RuO_2_-TiO_2_@Ti/PVDF membrane

Figure S2 shows the morphology and surface structure of the RuO_2_-TiO_2_@Ti electrode and RuO_2_-TiO_2_@Ti/PVDF membrane. It can be seen from Figure S2A that the RuO_2_-TiO_2_@Ti electrode consisted of numerous fine particles (RuO_2_ and TiO_2_), which was confirmed by the EDS and XRD analysis (see Figures S3, S4 and Table S1). The TiO_2_ nanoparticles displayed spherical shape with a diameter of 15-40 nm, while the RuO_2_ particles could be easily discerned in terms of their disparate color and relatively larger particle size (40-300 nm) than TiO_2_. Figure S2B indicates that the composite membrane has a typical structure of microfiltration membranes with an average pore size of 0.11±0.03 μm. Our experiments showed that the RuO_2_-TiO_2_@Ti/PVDF membrane and the original PVDF membrane had similar water-permeability performance with this finding in agreement with the results of previous works in which embedding electrodes into polymeric membranes had no adverse influence on the formation of membrane pores in PVDF active layer (Huang et al., 2015; Zheng et al., 2017).

**Figure 1:**
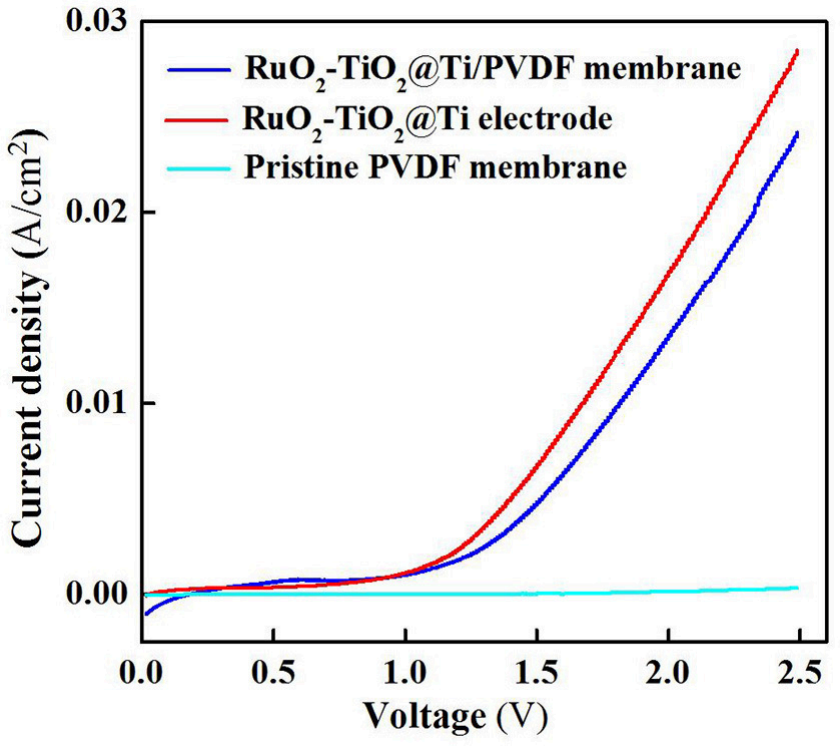
Linear sweep voltammetry curves of the RuO_2_-TiO_2_@Ti/PVDF membrane, RuO_2_-TiO_2_@Ti electrode and pristine PVDF membrane in the electrolyte solutions (pH = 7.0, *C*(PIPES) = 1 mM, *C*(Na_2_SO_4_) = 15 mM) at a potential scan rate of 10 mV/s. An Ag/AgCl and a Pt wire was used as the reference electrode and the counter electrode, respectively.

The electrochemical properties of the RuO_2_-TiO_2_@Ti-PVDF membrane and the RuO_2_-TiO_2_@Ti electrode were examined using linear sweep voltammetry (LSV) scan. As shown in Figure 1, the electrochemical composite membrane had similar current densities at lower potentials (<1.1 V) with the RuO_2_-TiO_2_@Ti anode, while the RuO_2_-TiO_2_@Ti anode performed slightly better at higher scanned potentials (>1.1 V), indicating that the casted membrane solution affected the conductivity of electrode though it still had favorable electrochemical properties (Zheng et al., 2017). The RuO_2_-TiO_2_@Ti electrode with a pore size of 100 μm is inefficient in intercepting suspended solids and colloids. Nonetheless, after coating with PVDF, the resultant RuO_2_-TiO_2_@Ti/PVDF composite membrane is expected to achieve simultaneous membrane separation (rejecting colloidal and particulate matter) and dissolved organic pollutant removal induced by electrochemical oxidation.

### Effect of operating conditions on electrochemical degradation of SA

**Figure 2:**
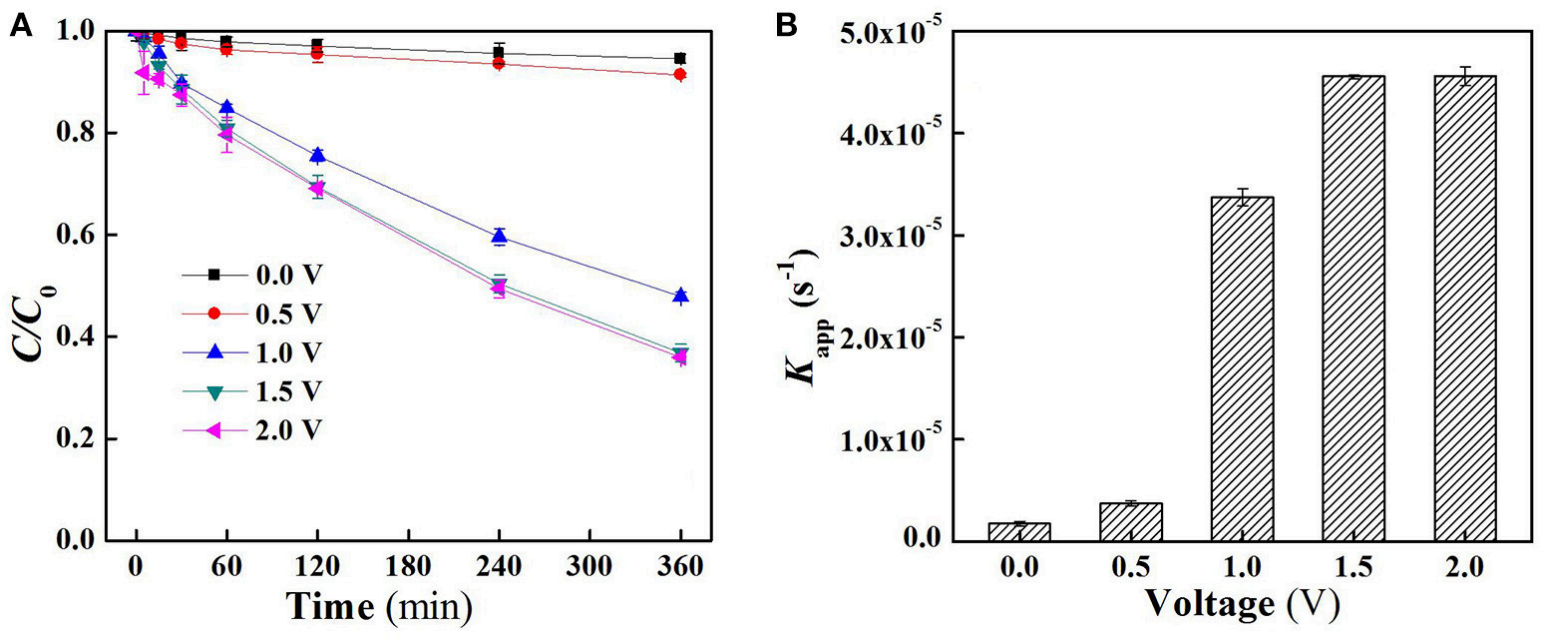
**(A)** Time course results of the proportion of aqueous SA remaining in the reactor following the application of different voltages under flow-by mode and **(B)** pseudo-first-order rate constants (*K*_app_) of SA degradation under flow-by mode. In plots **(A,B)**, pH = 7.0, *C*_0_ (SA) = 50 μM, *C*(PIPES) = 1 mM, *C*(Na_2_SO_4_) = 15 mM, applied voltage = 0, 0.5, 1.0, 1.5, and 2.0 V.

The charging voltage plays an important role in electrocatalytic reaction which governs the electron transfer rate and the generation of the specific oxidants (Jiang et al., 2010; Zhong et al., 2013). As shown in Figure 2A, there was almost no SA removal when low external electric field was applied (<1.0 V). Further increase in applied voltage resulted in an obvious degradation of SA, for instance, SA removal rate at 1.0 V and 1.5 V reached 52.1 and 63.2%, respectively, after 6 h operation under flow-by mode. However, the removal of SA was not further enhanced when a charging voltage higher than 1.5 V was applied (e.g., 2.0 V). This is mainly due to the fact that an exorbitant applied voltage could lead to intensified side reactions, e.g., water splitting, thus decreasing the current efficiency. Moreover, an excessively high voltage might also increase energy consumption and reduce the lifespan of electrodes (Niu et al., 2016). In all conditions, the degradation of SA follows pseudo-first-order kinetics. Further analysis of apparent rate constants (*K*_app_) for SA removal under flow-by mode (Figure 2B) shows that *K*_app_ at 1.5 V was 4.56 × 10^−5^ s^−1^ with this value about one order of magnitude higher than that obtained at 0.5 V (3.74 × 10^−6^ s^−1^).

**Figure 3:**
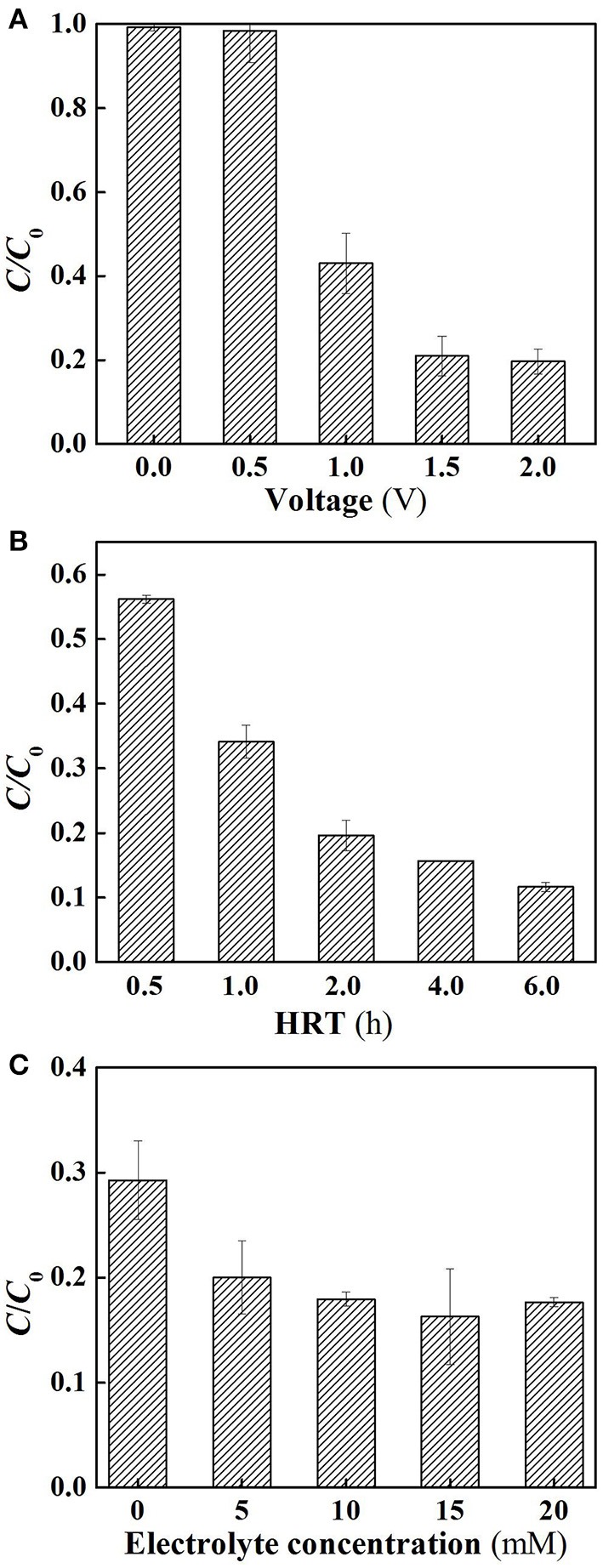
Electrochemical degradation of SA under flow-through mode. **(A)** Different applied voltages; **(B)** different HRT, and **(C)** different electrolyte concentrations. In plot **(A)**, pH = 7.0, *C*_0_ (SA) = 50 μM, *C*(PIPES) = 1 mM, *C*(Na_2_SO_4_) = 15 mM, HRT = 6 h, applied voltage = 0, 0.5, 1.0, 1.5, and 2.0 V. In plot **(B)**, pH = 7.0, *C*_0_ (SA) = 50 μM, *C*(PIPES) = 1 mM, *C*(Na_2_SO_4_) = 15 mM, applied voltage = 1.5 V, HRT = 0.5, 1, 2, 4, and 6 h; In plot **(C)**, pH = 7.0, *C*_0_ (SA) = 50 μM, *C*(PIPES) = 1 mM, applied voltage = 1.5 V, HRT = 2 h, *C*(Na_2_SO_4_) = 0, 5, 10, 15, and 20 mM.

Further experiments were performed in flow-through operated system in which the reactor was operated with the solution passing through the membrane at a constant membrane flux of 28.3 L m^−2^ h^−1^, yielding a hydraulic retention time (6 h) equivalent to that used in its flow-by operation. As shown in Figure 3A, under flow-through mode, the SA removal rates of the system increased as increasing charging voltages in the range between 0 and 1.5 V, similar to the tendency that was observed in the flow-by operated system. Further increase in the charging voltage up to 2.0 V gave rise to insignificant improvement of SA removal efficacy, again confirming the inefficiency of supplementary external electrical field. In view of the high removal rate at 1.5 V, subsequent experiments were carried out using this voltage unless otherwise stated. It is worth noting that compared to the SA degradation rates in the flow-by operated system, 4.9, 15.8, and 16.1% of increase in SA removal was observed in its flow-through operation at 1.0, 1.5, and 2.0 V, respectively. This is mainly attributed to the better contact of the contaminant with the oxidants generated in the vicinity of membrane surface under flow-through mode. Recent studies have shown that continuous flow operation can enhance mass transfer, stimulate the generation of ROS, and thereby facilitate the removal of target compounds (Zhao et al., 2013a; Li et al., 2016). This clearly demonstrates the advantage of integrating electrochemical oxidation into membrane filtration technology.

Figure 3B shows the influence of hydraulic retention time on SA degradation under flow-through mode. The SA removal rate increased from 43.8 to 80.4% as the HRT increased from 0.5 to 2 h. This may be because the HRT affected the effective contact time between catalytic membrane and SA solution. However, the SA removal rates were not dramatically increased when the HRT was further increased to 4 or 6 h. It is noteworthy that from the comparison of Figures 2A, 3B, at the charging voltage of 1.5 V, in order to attain a high SA removal rate (>60%), the required operating time for the flow-by operated system reached 6 h with this value significantly longer than that needed for its flow-through operation (HRT = 1 h). As such, the SA removal rates of different HRT under flow-through mode were found to be 25.2~49.8% higher than that of flow-by mode under comparable operating time. These results reveal that the space efficiency of the electrochemical system could be tremendously improved by flow-through operation as a consequence of enhanced mass transfer in this scenario.

The influence of electrolyte concentration on SA degradation is shown in Figure 3C. It can be observed that when the electrolyte concentration was higher than 10 mM, there was no significant influence of the electrolyte concentration on SA removal (*p* > 0.05). For lower concentrations (0 and 5 mM), a lower SA removal rate was observed, probably because a much lower concentrations of the electrolyte caused the increase in electric resistance of the solution, thus decreasing the oxidation efficiency.

### Electrochemical degradation mechanisms of SA

**Figure 4:**
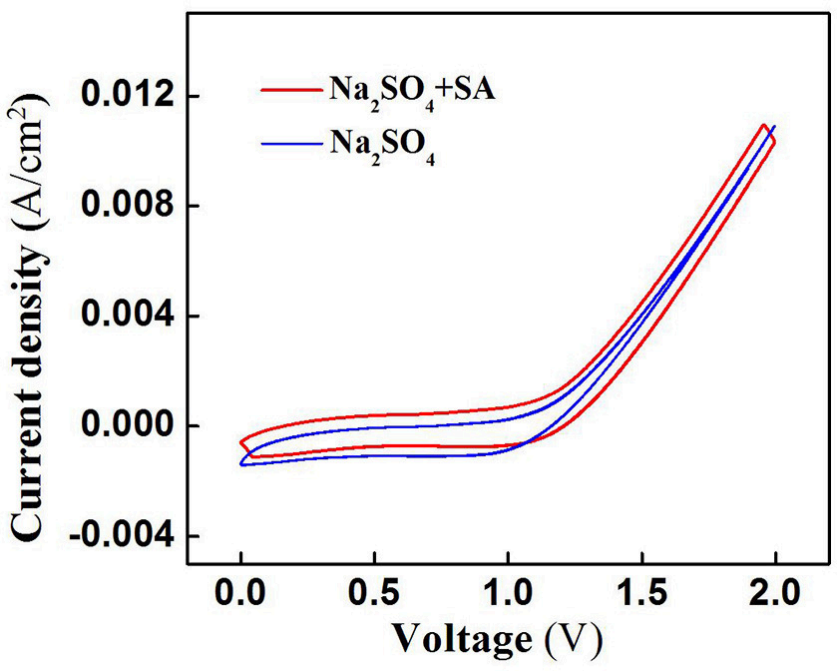
Cyclic voltammograms of the RuO_2_-TiO_2_@Ti/PVDF membrane in different electrolyte solutions (pH = 7.0, *C*_0_ (SA) = 0 or 50 μM, *C*(PIPES) = 1 mM, *C*(Na_2_SO_4_) = 15 mM) at a potential scan rate of 100 mV/s. An Ag/AgCl and a Pt wire was used as the reference electrode and the counter electrode, respectively.

SA degradation in the electrochemical system might be associated with two mechanisms: (i) ROS-mediated indirect oxidation initiated by H_2_O_2_, ${\text{HO}}_{2}^{•}$/${\text{O}}_{2}^{•-}$ and ^•^OH (Ramasundaram et al., 2013; Luo et al., 2014; Ammar et al., 2015; Sun et al., 2015), and/or (ii) direct oxidation via direct electron transfer of SA with the anode surface. To clarify the role of direct oxidation in SA decay, the cyclic voltammetry (CV) scan of RuO_2_-TiO_2_@Ti/PVDF membrane in the absence and presence of 50 μM SA was performed. It can be observed from Figure 4 that there was no obvious oxidation or reduction peak within scanning potential range of 0 to 2.0 V, suggesting that ROS-mediated oxidation was the dominant mechanism responsible for SA degradation rather than direct electron transfer (Yang et al., 2011, 2012).

**Figure 5:**
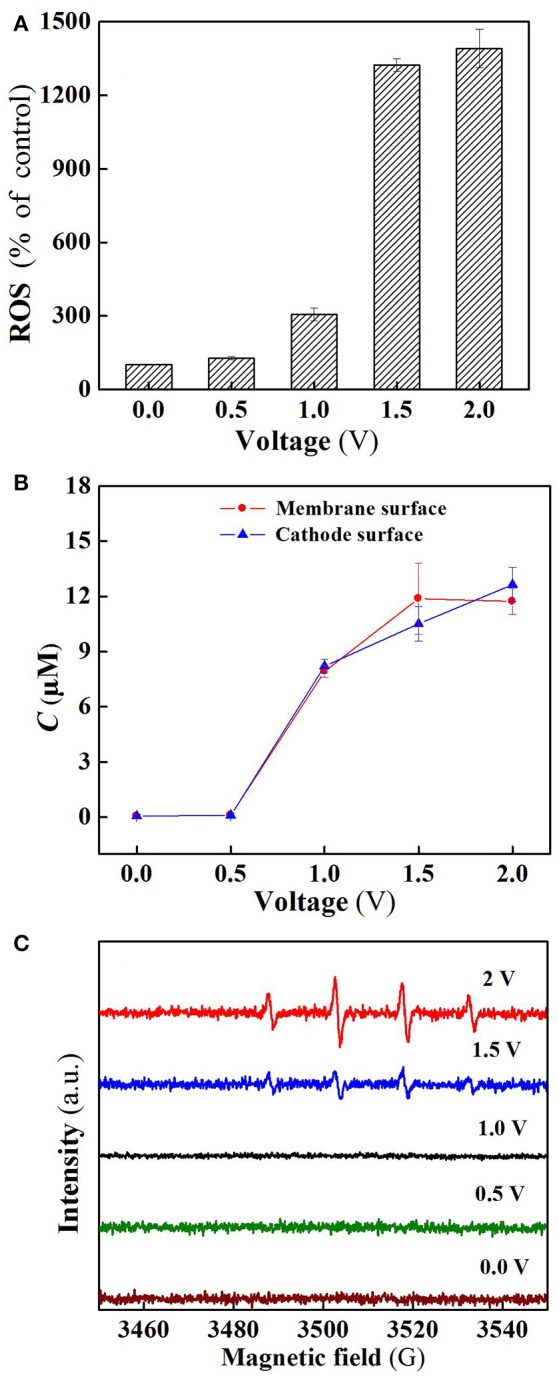
**(A)** ROS, **(B)** H_2_O_2_, and **(C)** DMPO spin-trapping ESR spectra (detecting ^•^OH) in the system at different voltages. In plots A-C, pH = 7.0, *C*(PIPES) = 1 mM, *C*(Na_2_SO_4_) = 15 mM, applied voltage = 0, 0.5, 1.0, 1.5, and 2.0 V.

As shown in Figure 5A, the ROS production in the flow-through operated system was increased with increasing applied voltages, for instance, the quantity of ROS at charging voltage 1.5 V was about 3 orders of magnitude higher than that of control. Furthermore, it is clear that the ROS quantity at 2.0 V was almost the same as that at 1.5 V, well explaining that SA removal rate was not significantly increased (Figure 3A) as the charging voltage was varied from 1.5 to 2.0 V.

Figure 5B reveals the production of H_2_O_2_ in the flow-through operated system under oxic condition at different charging voltages. It is obvious that H_2_O_2_ production in the system was also positively correlated with the applied electric field strength in the range of 0 to 1.5 V. As the membrane anode is electrified, excitation of TiO_2_ leads to the presence of holes in the valence band (*h*${}_{\text{vb}}^{+}$) and electrons in the conduction band (*e*${}_{\text{cb}}^{-}$) at the TiO_2_ surface (Equation 1). The generated *h*${}_{\text{vb}}^{+}$ and *e*${}_{\text{cb}}^{-}$ can not only decompose H_2_O into O_2_ and H_2_ (Equations 2 and 3) (Fujishima and Honda, 1972), but also react with the adsorbed H_2_O and O_2_ at the TiO_2_ surface to generate ROS such as ^•^OH, ${\text{O}}_{2}^{•-}$/${\text{HO}}_{2}^{•}$, and H_2_O_2_ that are potentially responsible for pollutants removal (Equations 4–9) (He et al., 2009). Beside this, in the co-doping of TiO_2_ and RuO_2_, RuO_2_ can serve as a conductor to transfer the excited *e*${}_{\text{cb}}^{-}$ of TiO_2_, thereby improving the separation efficiency of electron-hole pairs, and also participate in the reaction with surface adsorbed H_2_O to generate active ^•^OH (Equation 10) (Adhikari et al., 2018; Li et al., 2018a). In addition, the two-electron reduction of oxygen (Equation 11) also results in the generation of H_2_O_2_ at the cathode surface.

Electron spin resonance (ESR) analysis were further performed to detect ^•^OH production in the flow-through operated system at different applied voltages. As can be seen from Figure 5C, four characteristic peaks of DMPO-^•^OH were observed for the ESR spectra of 1.5 and 2.0 V, while no such signals were detected in the case of applied voltages below 1.5 V. Furthermore, the peak intensity for 2.0 V is much higher than that of 1.5 V, confirming that a larger amount of ^•^OH radicals was generated when high voltage (2.0 V) was applied. However, higher ^•^OH production gave rise to unobvious increase in SA removal (Figure 3A), likely due to the parasitic non-oxidizing reactions of ^•^OH (El-Ghenymy et al., 2012).

(1) $$\begin{array}{r} \text{Ti}{\text{O}}_{2}\to \text{Ti}{\text{O}}_{2}\left({h_{vb}}^{+}+{e_{cb}}^{-}\right) \end{array}$$

(2) $$\begin{array}{r} {\text{H}}_{2}\text{O}+2{h_{vb}}^{+}\to 0.5{\text{O}}_{2}+2{\text{H}}^{+} \end{array}$$

(3) $$\begin{array}{r} 2{\text{H}}^{+}+2{e_{cb}}^{-}\to {\text{H}}_{2} \end{array}$$

(4) $$\begin{array}{r} {h_{vb}}^{+}+{\text{H}}_{2}\text{O}\to^{•}\text{OH}+{\text{H}}^{+} \end{array}$$

(5) $$\begin{array}{r} {\text{O}}_{2}+{e_{cb}}^{-}\to {{\text{O}}_{2}{}^{•}}^{-} \end{array}$$

(6) $$\begin{array}{r} {{\text{O}}_{2}{}^{•}}^{-}+{\text{H}}^{+}\to {\text{H}{\text{O}}_{2}}^{•} \end{array}$$

(7) $$\begin{array}{r} {2\text{H}{\text{O}}_{2}}^{•}\to {\text{H}}_{2}{\text{O}}_{2}+{\text{O}}_{2} \end{array}$$

(8) $$\begin{array}{r} {e_{cb}}^{-}+{\text{H}}_{2}{\text{O}}_{2}\to^{•}\text{OH}+\text{O}{\text{H}}^{-} \end{array}$$

(9) $$\begin{array}{r} {\text{H}}_{2}{\text{O}}_{2}+{{\text{O}}_{2}{}^{•}}^{-}\to^{•}\text{OH}+\text{O}{\text{H}}^{-}+{\text{O}}_{2} \end{array}$$

(10) $${\text{H}}_{2}\text{O}{\overset{\text{RuO2}}{\to }}^{•}\text{OH}+{\text{H}}^{+}+e^{-}$$

(11) $$\begin{array}{r} {\text{O}}_{2}+2{\text{H}}^{+}+2e^{-}\to {\text{H}}_{2}{\text{O}}_{2} \end{array}$$

(12) $$\begin{array}{r} {\text{H}}_{2}{\text{O}}_{2}+\text{H}{{\text{O}}_{2}}^{-}\to^{•}\text{OH}+{{\text{O}}_{2}{}^{•}}^{-}+{\text{H}}_{2}\text{O} \end{array}$$

**Figure 6:**
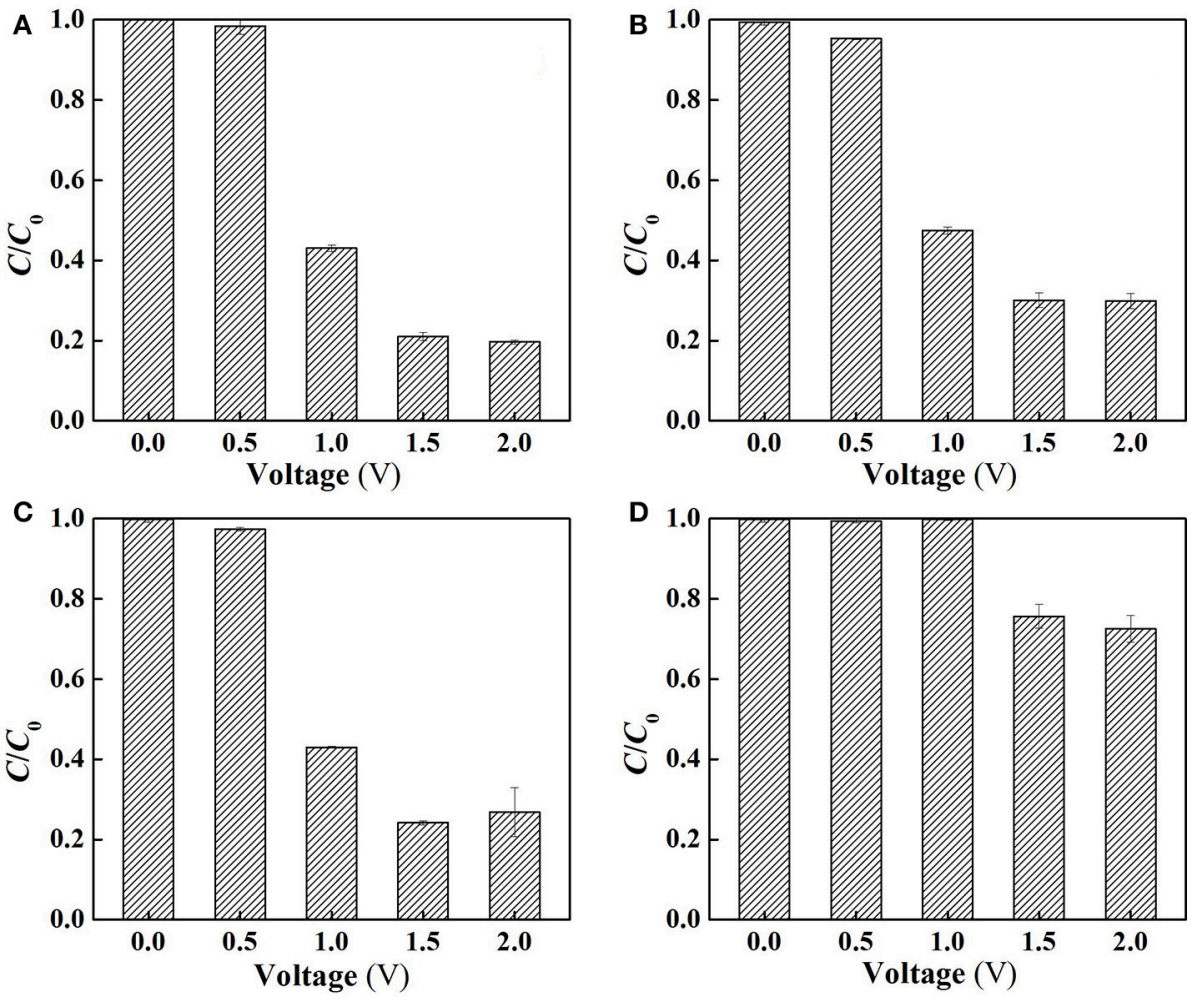
Electrochemical degradation of SA in the flow-through operated system at different applied voltages **(A)** under aerobic condition (air sparging), **(B)** under anoxic condition (N_2_ sparging), **(C)** following the addition of 2-propanol for dissociated ^•^OH quenching and **(D)** following the addition of TEMPOL for ${\text{O}}_{2}^{•-}$ quenching in the electrolyte solutions (pH = 7.0, *C*_0_(SA) = 50 μM, *C*(PIPES) = 1 mM, *C*(Na_2_SO_4_) = 15 mM, HRT = 2 h, applied voltage = 0, 0.5, 1.0, 1.5, and 2.0 V).

To clarify the specific roles of ROS in SA degradation, further experiments were performed at different charging voltages to assess the anoxic degradation behaviors of SA in the absence and presence of different scavengers (TEMPOL and 2-propanol for ${\text{O}}_{2}^{•-}$ and dissociated ^•^OH scavenging, respectively). The use of anoxic condition can exclude cathodic reactions (Equations 11, 12) by N_2_ sparging, and meanwhile ensure anodic oxidation to occur. As can be seen from Figure 6B, compared with the aerobic condition (Figure 6A), the removal rate of SA was decreased by 4.4, 9.1, and 10.2% at charging voltage of 1.0, 1.5, and 2.0 V, respectively. This indicates that the contribution of cathodic reactions in SA degradation was limited due to the inability to oxidize SA by H_2_O_2_ alone (Figure S5), though H_2_O_2_ might mediate production of small amount of ^•^OH at the cathode surface (Equation 12) (Martínez-Huitle et al., 2015).

Figure 6C demonstrates that the addition of 2-propanol resulted in merely 0, 3.2, and 7.2% of decrease in SA removal rate at 1.0, 1.5, and 2.0 V, respectively. This provides an interesting phenomenon, i.e., the dissociated ^•^OH that was diffused from the anode surface played a minor role in SA degradation. In contrast, the adsorbed ^•^OH on the anode (>^•^OH) that could not be quenched by 2-propanol played a more important role. It is also worth noting that dosing TEMPOL to quench ${\text{O}}_{2}^{•-}$ resulted in 52.8~56.8% decrease in SA removal rate (Figure 6D), suggesting that ${\text{O}}_{2}^{•-}$ made a great contribution to SA removal. However, in view of the relatively lower oxidation potential of ${\text{O}}_{2}^{•-}$ (1.3 V) than *E*^0^ (H_2_*O*_2_/*H*_2_O), the direct oxidation of SA by ${\text{O}}_{2}^{•-}$ was not likely to occur. Therefore, one can infer that ${\text{O}}_{2}^{•-}$, being an intermediate oxidant, played a dominant role in the production of ^•^OH.

**Figure 7:**
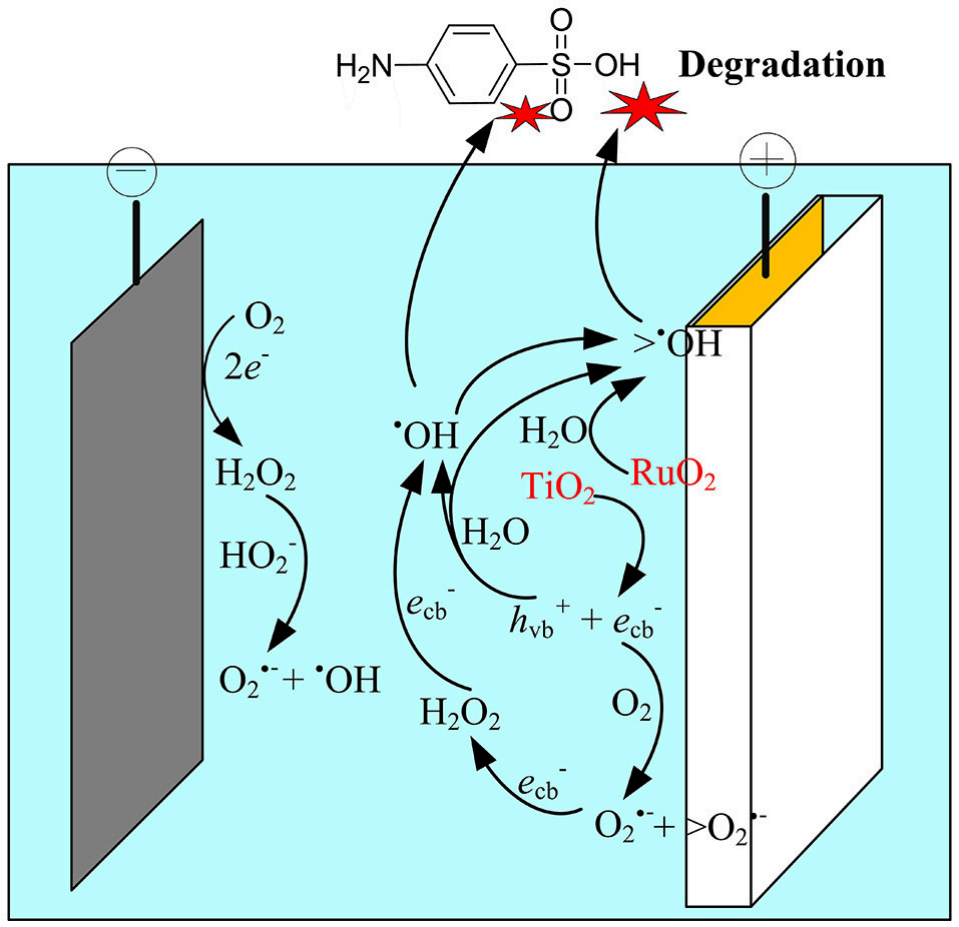
Schematic representation of the proposed mechanisms in the anodic electrochemical membrane filtration (EMF) system.

Based on the above analysis, the electrocatalytic mechanism for SA degradation in the EMF system was proposed (Figure 7). The main oxidation processes were assumed to occur at the anode surface, i.e., the polarization of anode could induce the excitation of TiO_2_, leading to production of holes in the valence band and electrons in the conduction band at TiO_2_ surface, and subsequently generation of ROS such as ^•^OH, ${\text{O}}_{2}^{•-}$/ ${\text{HO}}_{2}^{•}$, and H_2_O_2_. Meanwhile, the H_2_O molecule adsorbed on the surface of the RuO_2_ could lose electrons, being catalyzed to H^+^ and ^•^OH (Equation 10). Although indirect oxidation process was also observed at the cathode surface, it played a minor role in SA degradation in this system. The dominant oxidant responsible for SA degradation was determined to be the surface-adsorbed ^•^OH (>^•^OH), while ${\text{O}}_{2}^{•-}$ was an important intermediate oxidant involved in the production of ^•^OH.

### Enhanced oxidation efficiency of SA under flow-through mode

**Figure 8:**
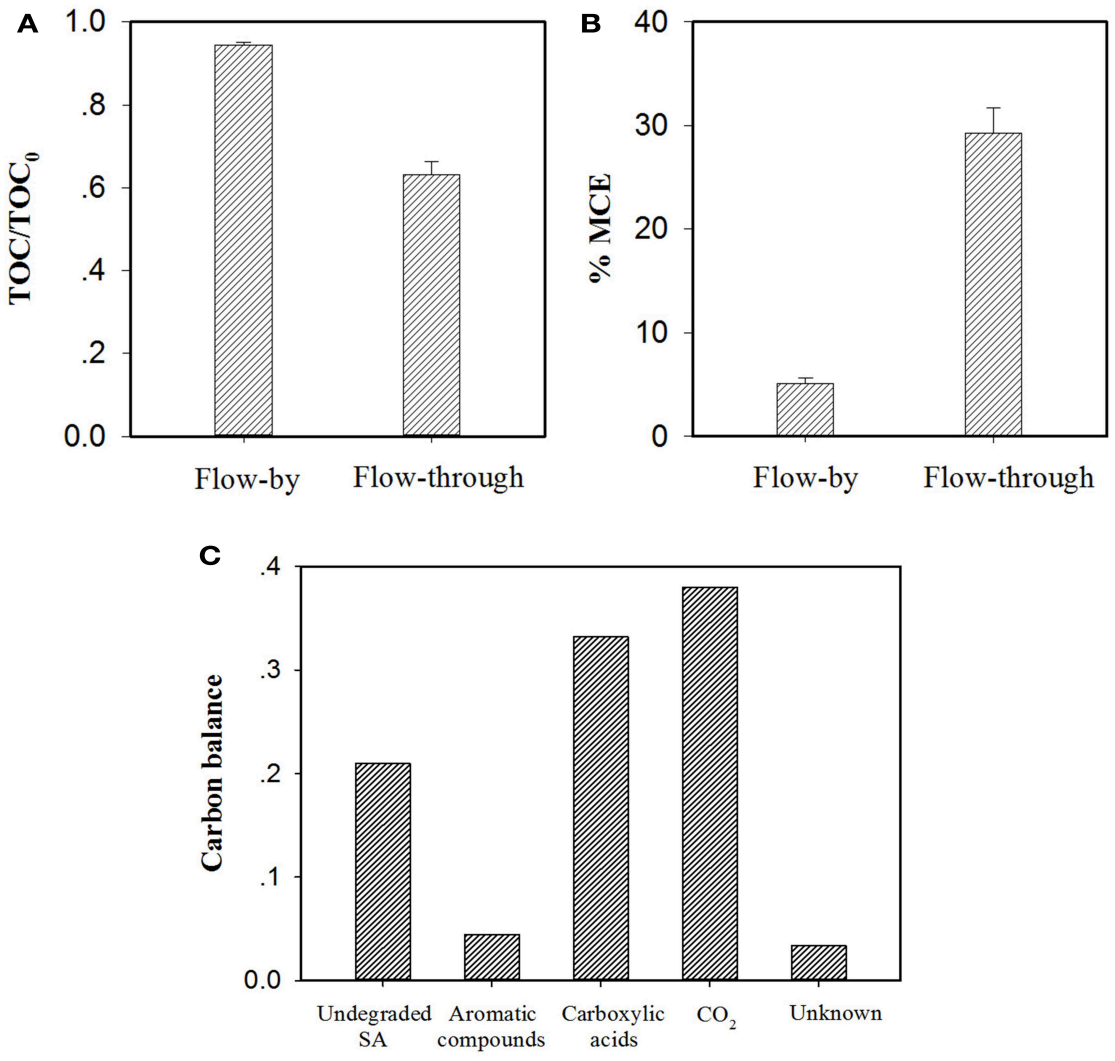
Comparison of **(A)** TOC abatement and **(B)** mineral current efficiency (MCE) for electrochemical treatment of a 50 μM SA solution (pH = 7.0, *C*(Na_2_SO_4_) = 15 mM, operating time/HRT = 2 h, applied voltage = 1.5 V) in the flow-by and flow-through operated system. The MCE values are calculated from Equation (S1). In particular, TOC determination experiments were performed with electrolyte solutions without adding PIPES, pH of the electrolyte solution was monitored by a pH meter and maintained at pH = 7.0 by adding aliquots of 0.1 M NaOH solution if necessary. **(C)** Mass balance on the basis of carbon stoichiometry using the flow-through operated system (HRT = 2 h). Aromatic compounds: hydroquinone and benzoquinone; Carboxylic acids: maleic, fumaric, oxamic, oxalic, formic and acetic acids. Proportion of Unknown was estimated by deducting the defined proportions (undegraded SA, aromatic compounds, carboxylic acids and CO_2_) from the total carbon.

As discussed earlier, in the EMF system, flow-through operation led to more efficacious SA elimination than its flow-by mode in all conditions. Additional experiments were carried out with consideration given to TOC abatement and mineral current efficiency (MCE) of the EMF system under different operating modes. As can be seen from Figure 8A, at the same charging voltage (1.5 V) and operating time/HRT (2 h), flow-through operation resulted in a TOC removal rate of 36.8%, much higher than that obtained in flow-by operation (5.5%). It is worth noting that the average current of the flow-through operated system was 6.1 mA with this value relatively higher than that of the flow-by one (5.2 mA), likely associated with the fast electron transfer reaction at the electrode/electrolyte interface under flow-through mode (Zhao et al., 2013a; Zheng et al., 2017; Li et al., 2018b), for instance, the accelerated transport of dissolved oxygen toward anode surface could benefit its one-electron reduction (Equation 5). The enhanced mass transfer might facilitate the production of ROS in the system as well. Moreover, in comparison to the calculated MCE value of the flow-by operated system (5.1%), a 5.7-fold increase in MCE value was observed in the flow-through operation (29.3%), as shown in Figure 8B, corroborating that flow-through operation can significantly improve the utilization efficiency of electric energy.

Recent studies have shown that the degradation of SA during electrooxidation processes generates organic byproducts (El-Ghenymy et al., 2012; Zheng et al., 2017). According to the RP-HPLC, IEC and/or GC analysis of the effluent extracted from the flow-through operated EMF system (HRT = 2 h, charging voltage = 1.5 V), the remaining organic components in the effluent including SA and its byproducts were identified and quantified with their characteristics and concentrations summarized in Table S2. It is clear that the resulting effluent contained aromatic compounds (e.g., hydroquinone and benzoquinone) and short-chain aliphatic carboxylic acids (mainly oxamic, oxalic, formic and acetic acids). Furthermore, the mass balance of carbon stoichiometry after 2 h of electrolysis was evaluated. As can be seen from Figure 8C, 33.2% of initial C was present in the effluent in the form of carboxylic acids, while the precursor compounds of carboxylic acids (i.e., aromatic compounds) merely accounted for 4.5% of initial C. In view of the fact that under flow-through mode, the majority of SA (equivalent to 71.2% of initial C) was ultimately converted into CO_2_ and nontoxic carboxylic acids, the EMF system developed in this study can be used as an alternative technique for purification of SA contaminated waters.

## Conclusions

RuO_2_-TiO_2_@Ti/PVDF composite membrane was successfully prepared through a sol-gel method and a phase inversion process. The TiO_2_ and RuO_2_ particles were dispersed on the surface of titanium mesh uniformly, and coating PVDF layer onto RuO_2_-TiO_2_@Ti electrode surface had no significant influence on the conductivity of resultant composite membrane. More importantly, RuO_2_-TiO_2_@Ti/PVDF composite membrane exhibited good electrocatalytic activity for SA elimination. At a charging voltage of 1.5 V and an electrolyte concentration of 15 mM, flow-through operation with a hydraulic retention time (HRT) of 2 h resulted in a high SA removal efficiency (80.4%). Cyclic voltammetry (CV) analysis indicated that the direct anodic oxidation played a minor role in the electrochemical oxidation of SA. Electron spin resonance (ESR) spectra demonstrated the production of ^•^OH in the EMF system. With the contribution of cathodic reactions excluded by N_2_ sparging, anodic generated ROS is more likely responsible for SA decay. Further scavenging test using 2-propanol to quench dissociated ^•^OH merely resulted in slight decrease in SA removal rates at charging voltages of 0.5~2.0 V, illustrating that the dissociated ^•^OH diffused from the anode surface played a minor role compared to adsorbed ^•^OH on the anode (>^•^OH). Furthermore, it was found that ${\text{O}}_{2}^{•-}$ was an important intermediate oxidant which mediates the production of ^•^OH. The calculated mineralization current efficiency (MCE) of the flow-through operated system was 29.3% with this value much higher than that of the flow-by one (5.1%), as expected from the improved contact reaction of contaminants with ROS present at the anode surface. Additionally, flow-through operation contributed to efficient oxidation of SA toward CO_2_ and nontoxic carboxylic acids which account for 71.2% of initial C.

## Author contributions

JZ and KY carried out the experiment and wrote the manuscript; ZWu, JZ, and ML participated in the material preparation; ZWa supervised all the experiments and proofread the manuscript; KY and JZ contributed to the discussion.

### Conflict of interest statement

The authors declare that the research was conducted in the absence of any commercial or financial relationships that could be construed as a potential conflict of interest.
