# Supplementary material for: Effective Removal of Sulfanilic Acid From Water Using a Low-Pressure Electrochemical RuO2-TiO2@Ti/PVDF Composite Membrane
Source: Front Chem. 2018 Sep 6;6:395. doi: 10.3389/fchem.2018.00395 (PMC6135927; doi:10.3389/fchem.2018.00395)
Supplement: Supplementary file 1 [file Table_1.DOCX]

Supporting Information：

Section S1 Calculation protocols for mineralization current efficiency (MCE)

At a certain applied current *I* (A) and a given operating/HRT *t* (h), the MCE value (%) of the system can be calculated by Eq. S1:

$MCE=\frac{\text{nFVΔ(TOC)}_{\exp}}{4.32\times10^{7}\text{mIt}}\times100$ (S1)

Where *F* is the Faraday constant (96487 C mol^−1^), *V* is the volume of electrolyte solution (L), *Δ(TOC)*_exp_ is TOC abatement in solution (mg L^−1^), 4.32 × 10^7^ is the homogenization factor (3600 s h^−1^ × 12000 mg of C mol^−1^ ), and *m* is the total number of carbon atoms in SA (6). In particular, the stoichiometric number of electrons (*n*) consumed per SA molecule was taken as 28, assuming that SA is completely mineralized to CO_2_, NH_4_^+^ and SO_4_^2-^ according to Eq. S2:

 (S2)

**Figure S1** A schematic diagram of the electrochemical membrane reactor.

1-DC power supplier; 2-Influent peristaltic pump; 3-Graphite cathode; 4-Composite membrane module (i.e., anode); 5- Effluent peristaltic pump; 6-Diffuser; 7-Air pump. The blue lines with arrows represent the water production line out of the reactor.


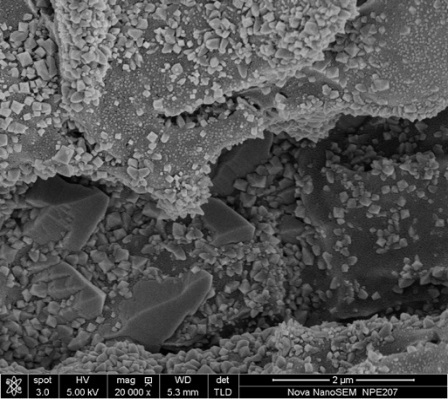

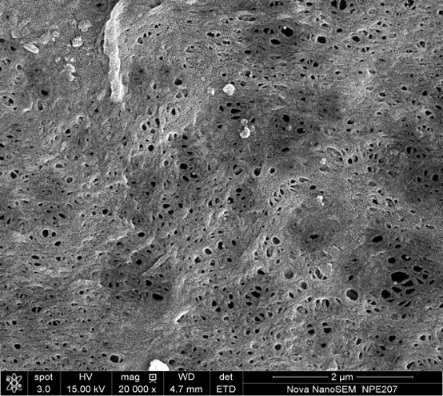


(A)

(B)

RuO_2_

TiO_2_

**Figure S2** SEM images of (A) the RuO_2_-TiO_2_@Ti electrode, and (B) the RuO_2_-TiO_2_@Ti/PVDF composite membrane

**Figure S3** EDS patterns of the RuO_2_-TiO_2_@Ti electrode

**Figure S4** XRD patterns of the RuO_2_–TiO_2_@Ti electrode

**Figure S5** Temporal variation of SA in the solution containing 20 *μ*M H_2_O_2_

**Table S1** The chemical composition of RuO_2_-TiO_2_@Ti electrode

| Element | *Wt*% | *Ar*% | |
| --- | --- | --- | --- |
| O K | 83.62 | 81.10 | |
| Ti K | 2.07 | 4.00 | |
| Ru L | 14.31 | 14.90 |  |

**Table S2** Characteristics and concentrations of remaining SA and organic byproducts of SA including aromatic compounds and short-linear carboxylic acids detected by RP-HPLC, IEC and/or GC in the extracted effluent of the flow-through operated EMF system (HRT = 2 h) in which a 50 *μ*M SA solution (pH = 7.0, *C*(Na_2_SO_4_) = 15 mM) was utilized as the influent and subject to 1.5 V of external electric field.

| Compound | Molecular formula | Analytical technique | Retention time  (min) | Molecular mass  (g mol^−1^) | Concentration  (*μ*M) |
| --- | --- | --- | --- | --- | --- |
| SA |  | RP-HPLC | 4.6 min | 173.20 | 10.49 |
| Hydroquinone |  | RP-HPLC | 7.2 min | 110 | 1.39 |
| Benzoquinone |  | RP-HPLC | 13.9 min | 118.09 | 0.84 |
| Maleic acid |  | IEC | 8.3 min | 116.07 | 1.89 |
| Fumaric acid |  | IEC | 14.2 min | 116.07 | 1.74 |
| Oxamic acid |  | IEC | 8.9 min | 89.05 | 3.96 |
| Oxalic acid |  | IEC | 6.7 min | 90.04 | 22.11 |
| Formic acid |  | IEC  GC | 13.3 min  5.8 min | 46.03 | 38.20 |
| Acetic acid |  | IEC  GC | 15.2min  4.7 min | 60.05 | 8.49 |
